# Utilization of Hyperspectral Imaging with Chemometrics to Assess Beef Maturity

**DOI:** 10.3390/foods12244500

**Published:** 2023-12-16

**Authors:** Simon A. Haughey, Holly Montgomery, Bernadette Moser, Natasha Logan, Christopher T. Elliott

**Affiliations:** 1 National Measurement Laboratory, Centre of Excellence in Agriculture and Food Integrity, Institute for Global Food Security, School of Biological Sciences, Queen’s University Belfast, Belfast BT9 5DL, UK; 2 Department of Chemistry, Institute of Analytical Chemistry, BOKU, University of Natural Resources and Life Sciences, Muthgasse 18, 1190 Vienna, Austria; 3 School of Food Science and Technology, Faculty of Science and Technology, Thammasat University, 99 Mhu 18, Pahonyothin Road, Khong Luang, Pathum Thani 12120, Thailand

**Keywords:** hyperspectral imaging, chemometrics, beef, maturity, classification model

## Abstract

There is a growing demand from consumers for more assurance in premium food products such as beef and especially steak. The quality of beef steak is primarily dictated by the maturation which ultimately influences its taste and flavor. These enhanced qualities have resulted in steak becoming a premium product that consumers are willing to pay a premium price for. A challenge, however, is analyzing the maturity of beef by traditional analytical techniques. Hyperspectral imaging (HSI) is a methodology that is gaining traction mainly due to miniaturization, improved optics, and software. In this study, HSI was applied to wet aged beef supplied at various stages of maturity, with spectral data generated using a portable hyperspectral camera. Two trials were conducted over a five-month period: (i) proof of principle and (ii) a bespoke sampling trial for the industry. With the support of industry participation, all samples were sourced from a highly reputable UK/Ireland supplier. To enhance data interpretation, the spectral data collected were combined with multivariate analysis. A range of chemometric models were generated using unsupervised and supervised methods to determine the maturity of the beef, and external validation was performed. The external validation showed good accuracy for “unknown samples” tested against the model set and ranged from 74 to 100% for the different stages of maturity (20, 30, and 40 days old). This study demonstrated that HSI can detect different maturity timepoints for beef samples, which could play an important role in solving some of the challenges that the industry faces with ensuring the authenticity of their products. This is the first time that portable HSI has been coupled with chemometric modeling for assessing the maturity of beef, and it can serve as a model for other food authenticity and quality applications.

## 1. Introduction

A crucial component in the modern meat industry is ensuring that consumer demand for premium products can be sustained by suppliers. Achieving this is underpinned by the sectors’ ability to evaluate the quality of meat with a view towards delivering a consistently premium product. In doing so, it justifies the cost associated with such products while providing the consumer with confidence in their purchase. Therefore, there is increasing interest in adopting technologies that can facilitate this process and quantify the key parameters and evaluation indicators in meat products.

For beef steak, the quality is usually indicated by its texture and flavor. This is achieved with fresh beef that has been dry or wet-aged from a few days to several weeks to allow the natural enzymes present to break down the muscle tissue. Therefore, aged beef commands a premium price on the open market, with consumers willing to pay extra for these enhanced qualities. The average retail price for a kilogram (kg) of steak in 2023 is GBP 16.45 (£), being the most expensive cut of beef on the market [1].

Over the past number of years, there has been rapid growth in the number of research publications using spectroscopy coupled with chemometric analysis to objectively measure different meat quality traits including moisture content, microbial count, species, and freshness [2,3,4,5,6,7,8]. More specifically, hyperspectral imaging (HSI) has been gaining traction due to being non-destructive, pollution-free, and having a rapid analysis time. These advantages are ideal for both the industry and research community. HSI can capture both the structural and biochemical information through the individual pixels collected across the wavelength range (400–1000 nm). Each pixel is broken into different spectral bands across the primary colors to provide a data cube of information. In a review article, Xiong et al. [9] emphasized advances in hardware and software developments of hyperspectral imaging systems, as well as discussing advances in using HSI to detect quality attributes of meat such as beef, lamb, and pork. The attributes highlighted as being measured using this technique included determination of color, pH, tenderness, water-holding capacity, microbial spoilage, and marbling, depending on the species of animal from which the meat was obtained. In a more recent review article, Jia et al. [10] outlined the use of HSI for meat quality evaluation across the supply chains, including its use in the following: (i) determining the color of fresh meat; (ii) determining the water content; (iii) determining the biogenic amine index; (iv) determining total viable count; (v) determining the nutritional composition; (vi) detecting adulteration; and (vii) determining gel and water injection. In addition, the review article also discussed possible future applications of HSI in the supply chain. However, there was no discussion around the application of HSI and chemometrics for determining beef maturity.

The adoption of chemometric modeling of the spectral data could be used to help observe any differences and/or similarities between variables in the data set. Two approaches can be employed in the model building method and include the use of unsupervised and supervised chemometric algorithms. The unsupervised method, principal component analysis (PCA), is usually employed initially to identify if there are any differences in the spectral data with no classifications applied. Supervised methods including partial least squares discriminant analysis (PLS-DA) and orthogonal partial least squares discriminant analysis (OPLS-DA) can be applied to help in assigning classes and can be used for predicting unknown samples against an authentic model set based on the input class description [11,12]. Therefore, chemometric analysis of spectral data plays an important role when using spectroscopic techniques and helps to model the differences found in the data.

In this study, collaboration with meat suppliers and processors in the UK/Ireland was vital to help understand the current testing conducted for meat quality. One of the challenges faced within the industry is determining the maturation of beef using a quick and simple analytical method. Therefore, the main aim of this study was to investigate if the maturation of sirloin beef steaks could be distinguished on different days of the aging process using HSI coupled with chemometrics. To the best of the authors knowledge, this is the first time that this combination of HSI and chemometrics has been used to determine the maturity of meat. Potentially, this could help in the food supply chain testing where food packagers rely solely on the paperwork supplied by the processors. Method development within this area would not only be beneficial to the industry but also to the consumer and would help to reduce waste.

## 2. Materials and Methods

### 2.1. Sample Collection

All samples were received from a commercial beef processor and selected randomly during normal plant operations. Samples were de-boned prior to being received and cut to size (each sample was less than 1 kg) to reduce wastage.

### 2.2. Scope of Methods

Over a five-month period during 2022, two trials were conceptualized and conducted for testing sirloin beef steaks for maturity. Testing was conducted over winter, spring, and summer to allow for seasonal variation to be included in the studies.

#### 2.2.1. Trial 1 (Proof of Principle)

Trial 1 was designed to initially evaluate and optimize the HSI Imager and to develop a method for testing the beef samples. Wet-aged sirloin steaks (n = 160) were collected for testing at the various stages of maturation (days: 10; 20; 30; 40). Samples were vacuum-packed (40 samples per pack) at the processing plant and stored at 4 °C for less than 24 h before testing. All samples were locally sourced beef; however, the breed and sex of the animals were unknown during the trial.

#### 2.2.2. Trial 2 (Bespoke Sampling Trial)

Trial 2 primarily focused on conducting a more detailed study which included consideration of parameters such as breed, category (heifer or steer), grade, and age (animals under 36 months old). This was achieved by the beef processor supplying full traceability for each steak supplied. The breed of animal was based on typical breeds in the UK/Ireland: Aberdeen Angus (n = 32), Charolais (n = 30), Limousin (n = 28), Holstein Friesian (or crosses) (n = 20), Hereford (n = 28), Simmental (n = 18), Shorthorn (n = 10), and British Blue (n = 20). The beef samples were taken from 70 heifers and 116 steers and were graded according to the EUROP classification standard [13]. The samples were assigned to four of the five different classifications for beef and included the following: Very Good (U) n = 34, Good (R) n = 72, Fair (O) n = 60, and Poor (P) n = 20. The grades given to the carcasses determine the value of the beef and classify both the conformation (shape) and fat cover of the carcass, which determines the price in pence per kilogram (p/kg) [14]. To allow for variation amongst animals, a maximum of one sample per loin (therefore, a maximum of 2 per animal) was taken and vacuum-packed. In total, 186 wet-aged sirloin steaks were collected for testing at 3 stages of maturation (days: 20, 30, and 40). All samples were received individually vacuum-packed and stored at 4 °C before their assigned day of testing.

### 2.3. Acquisition of Hyperspectral Images and Chemometric Analysis (Including Validation Methods)

**Figure 1:**
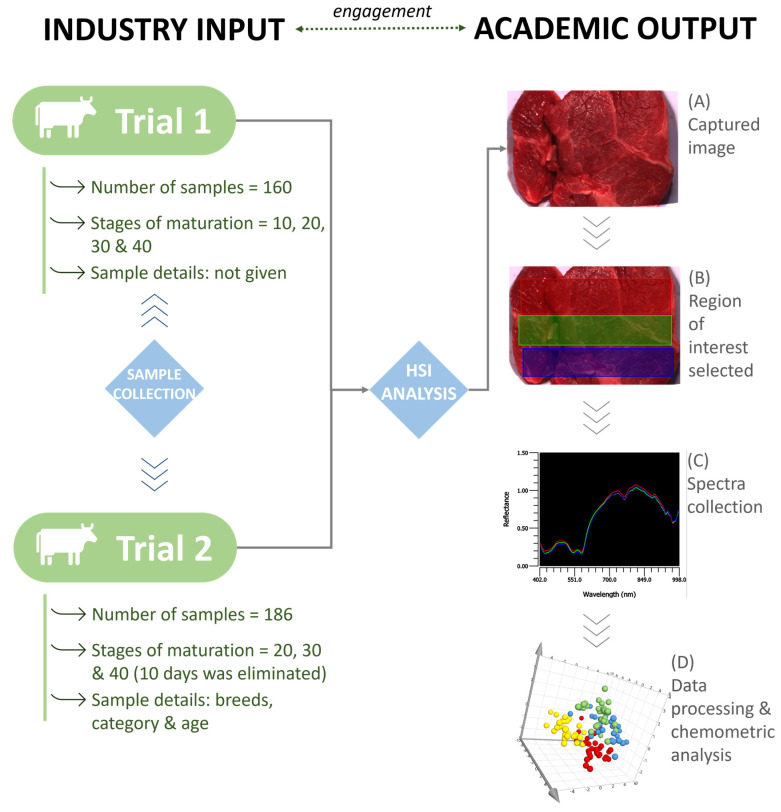
Instrument set-up and workflow for data acquisition and chemometric analysis.

No sample preparation was required prior to analysis, and samples were analyzed as received. Each sample was analyzed on a fixed platform using the portable HinaLea 4250 VNIR HSI camera (HinaLea Imaging, Emeryville, CA, USA). The working spectral range of the system was 400–1000 nm with 4 nm spectral resolution and 2.3 MegaPixel color sensor with 300 spectral wavelengths. In addition to the HSI camera, two adjacent halogen light bulbs were mounted on a stand for illumination of the samples. The camera settings were optimized to ascertain image quality. Both intensity and reflectance data were collected within the processing options, and the exposure was set to 32.2 ms and gain to 0. Prior to analysis, dark and white reference standards were used to calibrate the system using HinaLea Truscope Windows 1.1.17. One beef image (13 × 8 cm (l × b)) was obtained (Figure 1A) for each sample. For this work, the whole region of the collected image contained information of interest held within a three-dimensional hypercube (*x*, *y*, *λ*). The size of the beef samples in the acquisition of the image played an important role in trying to avoid any bias and selecting the regions of interest (ROI). Due to the variation in fat/muscle of the samples, it was decided to include the whole sample instead of selecting points of interest which may have introduced bias. Three ROIs were selected to capture data from the whole sample. ROIs were manually selected using the HSI software (version 1.1.17) and indicated by different colors: red, green, and blue (Figure 1B). Each sample was averaged prior to data processing (Figure 1C,D). Figure 1 provides a schematic overview of the workflow applied to generate the data and build the chemometric models.

The data were subsequently evaluated by chemometric analysis using SIMCA 17 software (Version 17.0.2.34594, Sartorius Data Analytics AB, Umea, Sweden). The raw data for both trials were treated in the same way using the following steps of pre-processing, model building (with internal cross-validation), and external validation. Validation was performed based on the procedure described and recommended by the US Pharmacopoeia [15] and McGrath et al. [16].

## 3. Results and Discussion

### 3.1. Trial 1

#### 3.1.1. Spectra Collection

**Figure 2:**
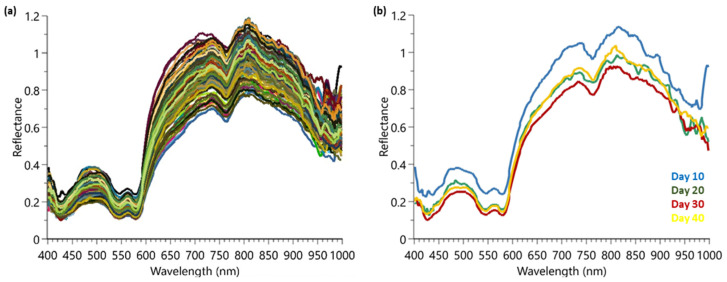
HSI spectra collected from Trial 1 for maturation determination: (**a**) complete raw data set showing the 160 samples; (**b**) the averaged spectrum for a sample from day 10, 20, 30, and 40.

After analysis of the beef samples, all spectra were collected and extracted from the HSI software. Figure 2a shows the raw data spectra which were collected prior to any pre-processing filters. Figure 2b shows the averaged spectra of the samples at the different maturation time points (10, 20, 30, and 40 days).

Visually, the reflectance spectral data for the four time points are extremely similar, with some distribution variation around 800–1000 nm between samples. It is important to use all wavelengths available since in a previous publication, the authors indicated that reduced wavelength models using only the most important wavelengths showed reduced and erratic levels of performance [17]. To aid in the spectra interpretation, chemometrics were employed for modeling.

#### 3.1.2. Chemometric Modeling

**Figure 3:**
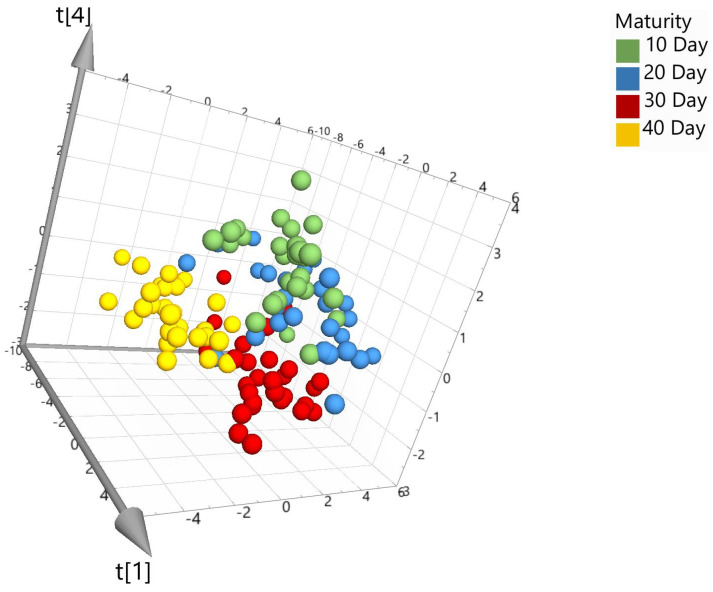
PCA 3D score plot for the model set colored according to maturity days (where the *t* axis refers to the models’ vector for each dimension and is based on new variables as linear combinations of X).

To examine the hyperspectral data, spectra were transferred to SIMCA 17 chemometric software to aid in multivariate data analysis. Prior to model building, several pre-processing steps were applied to the data set to assist in eliminating background noise, light scattering, and sample texture. Five different types of pre-processing filters were applied to the spectra data and included the following: standard normal variate (SNV), first derivative (1st D), second derivative (2nd D), Savitzky–Golay (SG) smoothing, and pareto scaling (par). SNV was used to remove any slope differences in the spectra which may have occurred from light scattering. The software does this by subtracting the row mean and dividing it by the row standard deviation. SG was also applied for noise canceling and smoothing of the data set. First or second derivatives were applied to transform the original data set to its first or second derivatives which can help in removing any peak overlap and baseline drift. Lastly, pareto scaling was used which divides each variable by the square root of its standard deviation [18]. In this study, principal component analysis (PCA), partial least square discriminant analysis (PLS-DA), and orthogonal projections to latent structures discrimination analysis (OPLS-DA) were applied for model building. Models can be visually viewed within the software using 2D or 3D score scatter plots. Figure 3 shows the unsupervised PCA score plot (SNV; pareto scaling). Visually, the PCA plot shows limited separation between the different maturation dates and therefore requires further modeling using supervised algorithms, PLS-DA and OPLS-DA.

#### 3.1.3. Internal Validation of Chemometric Models

Internal validation was performed by the software for all of the models, and Table 1 provides an overview of the supervised calibration models (including pre-processing) and the predictive values based on internal cross-validation for the data set.

The R2 values relate to the measure of the model’s fit to the original data. The range is from zero to one, with values closer to one indicating a good model fit. The R2 value is calculated using the following equation: R2 = 1 − RSS/SSX, where SSX is the overall sum of squares and RRS is the residual sum of squares = ∑(observed − fitted)^2^. The software performs internal cross-validation by removing 1/7th of the data and predicting it against a new model made from 6/7th of the remaining data. This process is repeated until all of the data have been predicted. The result is provided by the predicted residual sum of squares (PRESS), where the squared differences between the observed and predicted values for the data are excluded from the model fit, PRESS = ∑(y_im_ − ŷ_im_) [18]. In Table 1, model 14 (PLS-DA, with no pre-processing) shows the highest R2 value of 0.991. The Q2 value is provided from internal cross-validation based on the variation between X or Y that can be predicted by a component and can be calculated using the following: Q2 = 1 ∑ PRESS/SS, where SS is the sum of squares of Y [18]. The closer the value is to one, the greater the predictive power based on the internal validation. The supervised OPLS-DA model 8 has the highest Q2 value of 0.915, as shown in Table 1.

#### 3.1.4. External Validation of Calibration Models

**Figure 4:**
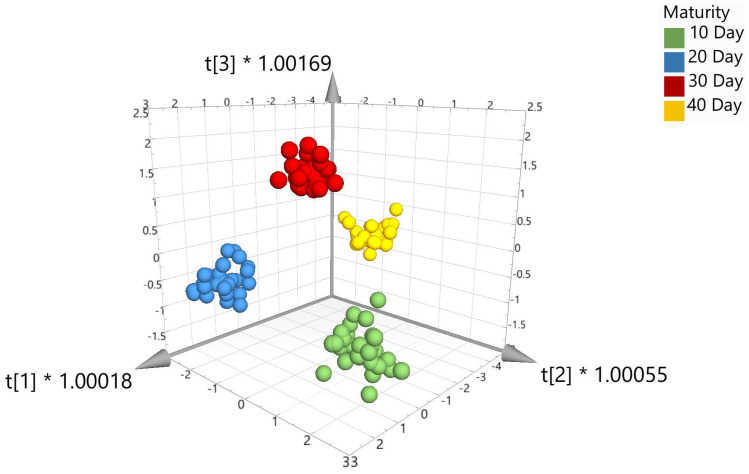
3D score scatter plot for OPLS-DA with SNV (model 8).

External validation was performed on the data set and included splitting the data 70:30, i.e., 112 samples (or 70% of the total number of samples) were randomly assigned to the calibration set and 48 samples (or 30% of the total number of samples) were randomly assigned to the validation set. To create the validation set, each time point (10, 20, 30, and 40 day) was randomized (using Excel), and then, 70% of the samples were taken for the calibration set and the remaining samples were added to the validation set. The calibration sample set was representative of correctly identified beef samples for the four different time points. The validation set was composed of an equal mix of correctly identified samples that do not form part of the calibration set. The validation set was tested against the calibration set and used to generate the model. Prediction percentages were generated and could be assigned to the relevant classes as shown in Table 2. Overall, the 12 calibration models in Table 2 show high percentages for correctly predicting and identifying external meat samples matured over 10, 20, 30, and 40 days. Five of the models show that 100% of the meat samples were correctly identified across the four ageing time points (highlighted in green). As an example of a supervised chemometric model, Figure 4 shows the visual 3D score scatter plot for model 8 with the different maturity classes which are color coded in green (10-day maturity), blue (20-day maturity), red (30-day maturity), and yellow (40-day maturity). The model shows each of the maturity groups clustering tightly together and major separation between the four maturity classes. Table 3 shows the confusion matrix for the validation set tested against the model set.

Table 3 highlights the days that the beef matured over 10, 30, and 40 days could be correctly identified, with only two samples from day 20 incorrectly assigned as day 10 and day 40. From this confusion matrix, different performance metrics can be evaluated, i.e., accuracy, specificity, recall/sensitivity, precision, f-score, and error rate, which will indicate the effectiveness of the model as recommended by Sokolova and Lapalme [19]. The average values found for each metric were as follows: (i) accuracy = 0.96; (ii) specificity = 0.99; (iii) recall/sensitivity = 0.94; (iv) precision = 0.96; (v) f-score = 0.95; and (vi) error rate = 0.08. Overall, these metrics, based on the results from Trial 1, indicate that the model performed extremely well and was fit for purpose, with a high number (96%) of the beef samples collected and analyzed being properly assigned to the correct maturity class.

### 3.2. Trial 2

During Trial 1, there were limited meta-data for the samples provided; therefore, it was unclear whether the samples had come from different carcasses, breeds, grades, or sex. Therefore, it was proposed for Trial 2 to gather more meta-data to improve the diversity of the samples collected for analysis. This would allow for a more bespoke model for the industry which would also help improve the reliability and robustness of the models created. In total, 186 samples were received for testing, with 62 samples at each of the maturation stages (20, 30, and 40 days). For Trial 2, it was decided not to include day 10, as sirloin steaks would not commonly be aged for only 10 days. Table 4 shows the breakdown of different breeds and sexes (i.e., heifers or steers) from which the samples were collected. These breeds were selected as they are the most typical breeds found in the UK and Ireland, with Aberdeen Angus being the most popular. Heifers and steers refer to female and male bovine, respectively, which have not been used to birth calves or reproduce. As a result, the meat produced by heifers and steers is normally of higher quality.

#### 3.2.1. Internal Validation of Calibration Models

**Figure 5:**
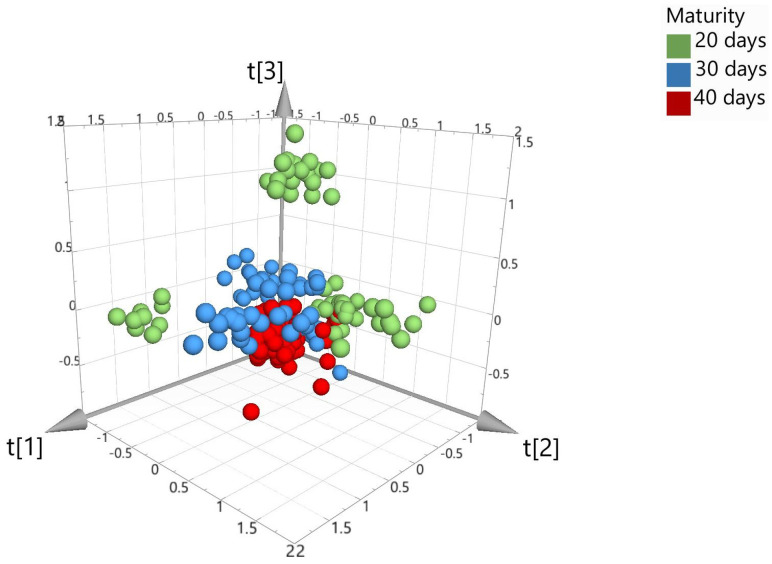
PCA 3D score plot, colored according to the number of days that beef was matured (model 3).

Chemometric models were created for the data acquired during Trial 2 using PCA, PLS-DA, and OPLS-DA algorithms. Figure 5 shows the unsupervised PCA score plot (with SNV + 1st Derivative + Savitzky–Golay; pareto scaling). As anticipated from Trial 1, visually, there was some limited separation across the three maturity classes using the unsupervised PCA algorithm. Therefore, further processing was required using the supervised algorithms, i.e., PLS-DA or OPLS-DA. 

**Figure 6:**
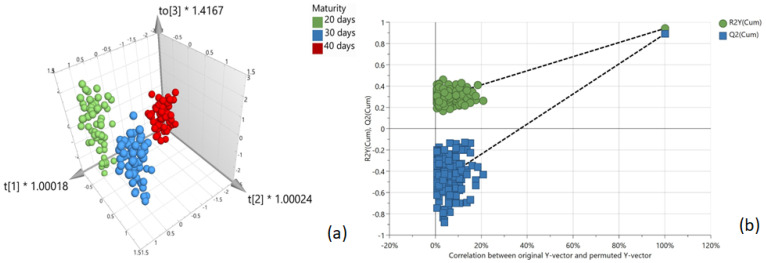
(**a**) OPLS-DA 3D score plot; (**b**) permutation analysis (n = 200) to test calibration model for overfitting the data.

Table 5 summarizes the model fit and predictive parameters achieved for the supervised models created (including details of the pre-processing techniques applied). The OPLS-DA model (M13), shown in Figure 6a, provided the highest R2 value of 0.99 (model fit) and a Q2 value of 0.852 (predictive measurement) which indicated that the generated calibration model could sufficiently determine the maturity or age of beef. To illustrate that the OPLS-DA model was not overfitting the data, which can lead to false-positive results, the model was further validated by performing a permutation test (200 times). The result of the permutation analysis in Figure 6b shows the correlation coefficients between original Y and permuted Y in relation to a cumulative R2 and Q2 value, with the *X*-axis displaying the correlation and the *Y*-axis showing the R2/Q2 values. Also displayed are fitted regression lines connecting the observed Q2 to the corresponding centroid of the permuted Q2 clusters. A model is considered valid if its Q2 values are higher than the permutation test values (i.e., all Q2 values of the permutated data set to the left are lower than the Q2 values of the original data set to the right), and, in addition, if a negative intercept value on the *Y*-axis is found for the regression line [20]. Therefore, based on the data generated from this permutation test, it can be determined that the OPLS-DA model does not overfit the data and the model is fit for purpose.

#### 3.2.2. External Validation of Calibration Models

As conducted during Trial 1, external validation was also performed on the data set in which the work set was split 70:30 (i.e., 129 samples were randomly assigned to the calibration sample set and 57 samples to the validation sample set). The validation set was then tested against the calibration set and prediction percentages were assigned to the relevant classes, as shown in Table 6.

Overall, the prediction results from the external validation for all 12 models and meat samples matured over 20, 30, or 40 days ranged from 74% to 100%. Day 20 showed the highest number of samples correctly assigned, with six models able to correctly match 100% of the meat samples. The highest percentage of meat samples correctly predicted for day 30 and day 40 was 84% and 95%, respectively.

**Figure 7:**
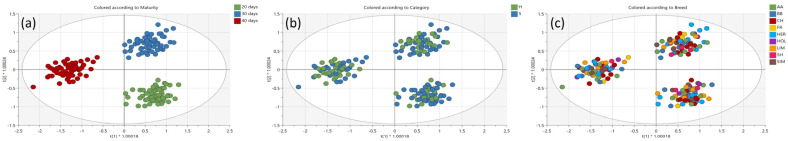
The 2D score plots of the OPLS-DA model (no pre-processing; pareto scaling) with classes colored by (**a**) maturity, (**b**) sex (heifer or steer), and (**c**) breed.

The calibration model (M17) shown in Figure 7a indicates that each of the maturity groups clustered together within their classes for days 20 (green), 30 (blue)), and 40 (red). Table 7 shows the confusion matrix for the validation set results which was tested against the model set. From the results, only one sample was incorrectly predicted for day 20 and day 40, and three samples were incorrectly predicted for day 30. As determined in Trial 1, average performance metrics were obtained for the Trial 2 results and were found to be as follows: (i) accuracy = 0.91; (ii) specificity = 0.96; (iii) recall/sensitivity = 0.91; (iv) precision = 0.91; (v) f-score = 0.91; and (vi) error rate = 0.18. Overall, the percentage of correctly identified samples for Trial 2 was 91%, which is lower than that achieved during Trial 1 (96%) and is attributable to the use of a more diverse sample set including different breeds of animals, different sex, and different seasons. However, these results, with very good performance metrics, indicate the potential of portable HSI coupled with chemometrics to determine the maturity of wet-aged beef. This technology could have a beneficial impact for the industry which may have limited or no procedures in place to test beef aging.

Due to the additional meta-data received during this phase of the study, further investigations were conducted to establish whether the breed or sex of the cattle (i.e., heifer or steer) could have a direct influence on the models developed. Therefore, the model was color coded by breed and sex to establish whether the original clustering observed was solely due to the maturity of beef samples. These results are highlighted in Figure 7b, whereby the scatter plot relates only to heifer or steer, and Figure 7c, which relates to cattle by breed. Overall, these results confirm that the calibration model was not influenced by the sex or breed of the cattle used in the study and was exclusively due to the maturity/age of the beef.

## 4. Conclusions

The adoption of innovative analytical methods is an important area for the meat industry. These developments help to maintain the quality and authenticity of meat products and ensure that consumers are consistently provided with high standard products, in addition to protecting the reputation of businesses. This study reports the utilization of portable HSI combined with chemometrics to successfully determine the maturity of sirloin steaks which ranged from 10 to 40 days aged. For both trials, the external validation sets could predict “unknown” samples, with 96% and 91% of meat samples correctly assigned to the correct maturity class during Trial 1 and Trial 2, respectively. The results highlight the potential for this non-destructive and rapid methodology to be employed as an authenticity tool within industry. Future research could also incorporate other parameters (including different cuts of meat) to develop further models and explore how this technique could be incorporated within a digitally connected food supply chain.

## Figures and Tables

**Table 1:** Overview of supervised models generated for Trial 1.

Model Number	Type (with Details of Pre-Processing Applied)	R2	Q2
M3	PLS-DA (with SNV; par scaling)	0.926	0.914
M4	PLS-DA (with SNV, 1st D, SG, and par scaling)	0.832	0.882
M5	PLS-DA (with SNV, 2nd D, SG, and par scaling)	0.796	0.888
M6	PLS-DA (with 1st D, SG, SNV, and par scaling)	0.805	0.88
M7	PLS-DA (with 2nd D, SG, SNV, and par scaling)	0.776	0.874
M14	PLS-DA (no processing; par scaling)	0.991	0.877
M8	OPLS-DA (with SNV; par scaling)	0.926	0.915
M9	OPLS-DA (with SNV, 1st D, SG, and par scaling)	0.736	0.865
M10	OPLS-DA (with SNV, 2nd D, SG, and par scaling)	0.779	0.883
M11	OPLS-DA (1st D, SG, SNV, and par scaling)	0.806	0.882
M12	OPLS-DA (2nd D, SG, SNV, and par scaling)	0.757	0.882
M13	OPLS-DA (no processing; par scaling)	0.982	0.8

**Table 2:** % of correct predictions from the validation sample set (results based on SIMCA classification results).

Model Number	Model	% of Correct Classification			
		For 10 Days	For 20 Days	For 30 Days	For 40 Days
M6	PLS-DA (with 1st D, SG, and SNV)	100.0	100.0	100.0	100.0
M7	PLS-DA (with 2nd D, SG, and SNV)	100.0	100.0	100.0	100.0
M4	PLS-DA (with SNV, 1st D, and SG)	100.0	100.0	100.0	91.7
M5	PLS-DA (with SNV, 2nd D, and SG)	100.0	91.7	100.0	100.0
M3	PLS-DA (with SNV)	100.0	83.3	100.0	100.0
M14	PLS-DA (no processing)	100.0	83.3	100.0	100.0
M9	OPLS-DA (with SNV, 1st D, and SG)	100.0	100.0	100.0	100.0
M10	OPLS-DA (with SNV, 2nd D, and SG)	100.0	100.0	100.0	100.0
M11	OPLS-DA (1st D, SG, and SNV)	100.0	100.0	100.0	100.0
M12	OPLS-DA (2nd D, SG, and SNV)	100.0	100.0	91.7	100.0
M8	OPLS-DA (with SNV)	100.0	83.3	100.0	100.0
M13	OPLS-DA (no processing)	91.7	66.7	83.3	91.7

**Table 3:** Confusion matrix based on validation data determined using model 8.

		Predicted Class				False Negatives
		Day 10	Day 20	Day 30	Day 40	
True Class	Day 10	12	0	0	0	0
	Day 20	1	10	0	1	2
	Day 30	0	0	12	0	0
	Day 40	0	0	0	12	0
	False Positives	1	0	0	1	

**Table 4:** Breakdown of sample breeds and sex of animals included in Trial 2.

Breed	No. of Each Breed	Heifer	Steer
Aberdeen Angus (AA)	32	14	18
Charolais (CH)	30	18	12
Limousin (LIM)	28	14	14
British Blue (BB)	20	8	12
Simmental (SIM)	18	8	10
Holstein (HOL)	12	-	12
Shorthorn (SH)	10	-	10
Friesian (FR)	8	-	8
Total	186	70	116

**Table 5:** Overview of models (including pre-processing details) created for Trial 2.

Model Number	Type (with Details of Processing Applied)	R2	Q2
M7	PLS-DA (no processing; par scaling)	0.989	0.749
M8	PLS-DA (SNV; par scaling)	0.929	0.763
M9	PLS-DA (SNV + 1st Der + SG; par scaling)	0.802	0.734
M10	PLS-DA (SNV + 2nd Der + SG; par scaling)	0.745	0.784
M11	PLS-DA (1st Der + SG + SNV; par scaling)	0.826	0.757
M12	PLS-DA (2nd Der + SG + SNV; par scaling)	0.706	0.794
M13	OPLS-DA (no processing; par scaling)	0.99	0.852
M14	OPLS-DA (SNV; par scaling)	0.912	0.802
M15	OPLS-DA (SNV + 1st Der + SG; par scaling)	0.761	0.709
M16	OPLS-DA (SNV + 2nd Der + SG; par scaling)	0.744	0.806
M17	OPLS-DA (1st Der + S-G + SNV; par scaling)	0.825	0.779
M18	OPLS-DA (2nd Der + S-G + SNV; par scaling)	0.704	0.822

**Table 6:** % of correctly identified predictions from the validation sample set.

Model Number	Model	% of Correct Classification		
		For 20 Days	For 30 Days	For 40 Days
M7	PLS-DA (no processing; par scaling)	100	84	89
M8	PLS-DA (SNV; par scaling)	100	74	89
M9	PLS-DA (SNV + 1st Der + SG; par scaling)	100	79	95
M10	PLS-DA (SNV + 2nd Der + SG; par scaling)	79	74	74
M11	PLS-DA (1st Der + SG + SNV; par scaling)	95	79	84
M12	PLS-DA (2nd Der + SG + SNV; par scaling)	89	84	79
M13	OPLS-DA (no processing; par scaling)	100	74	89
M18	OPLS-DA (SNV; par scaling)	100	74	89
M14	OPLS-DA (SNV + 1st Der + SG; par scaling)	100	84	74
M15	OPLS-DA (SNV + 2nd Der + SG; par scaling)	95	79	100
M16	OPLS-DA (1st Der + SG + SNV; par scaling)	95	79	84
M17	OPLS-DA (2nd Der + SG + SNV; par scaling)	95	84	95

**Table 7:** Confusion matrix based on validation data determined using model 17.

		Predicted Class			False Negatives
		Day 20	Day 30	Day 40	
True Class	Day 20	18	1	0	1
	Day 30	1	16	2	3
	Day40	1	0	18	1
False Positives		2	1	2	

## Data Availability

Data are available on request due to restrictions. The data presented in this study are available on request from the corresponding author. The data are not publicly available due to possible future commercialization of the analytical test.

## Collaboration Statement

Co-author, Bernadette Moser, was a visiting student from BOKU. As part of her studies at Queen’s University Belfast, she worked on the project and contributed to its successful outcome.
